# Titin‐truncating variants are associated with heart failure events in patients with left ventricular non‐compaction cardiomyopathy

**DOI:** 10.1002/clc.23172

**Published:** 2019-04-16

**Authors:** Shijie Li, Ce Zhang, Nana Liu, Hui Bai, Cuihong Hou, Lei Song, Jielin Pu

**Affiliations:** ^1^ State Key Laboratory of Cardiovascular Disease, Fuwai Hospital, National Center for Cardiovascular Diseases Chinese Academy of Medical Sciences and Peking Union Medical College Beijing China; ^2^ Department of Cardiology Fuwai Hospital, National Center for Cardiovascular Disease, Chinese Academy of Medical Sciences and Peking Union Medical College Beijing China; ^3^ Department of Cellular and Molecular Medicine Lerner Research Institute, Cleveland Clinic Cleveland Ohio; ^4^ Department of Cardiology Shanghai East Hospital, Tongji University Shanghai China

**Keywords:** genotype, left ventricular non‐compaction cardiomyopathy, phenotype

## Abstract

**Background:**

Titin‐truncating variants (TTNtv) have been recognized as the most prevalent genetic cause of dilated cardiomyopathy. However, their effects on phenotypes of left ventricular non‐compaction cardiomyopathy (LVNC) remain largely unknown.

**Hypothesis:**

The presence of TTNtv may have an effect on the phenotype of LVNC.

**Methods:**

*TTN* was comprehensively screened by targeted sequencing in a cohort of 83 adult patients with LVNC. Baseline and follow‐up data of all participants were collected. The primary endpoint was a composite of death and heart transplantation. The secondary endpoint was heart failure (HF) events, a composite of HF‐related death, heart transplantation, and HF hospitalization.

**Results:**

Overall, 13 TTNtv were identified in 13 patients, with 9 TTNtv located in the A‐band of titin. There was no significant difference in baseline characteristics between patients with and without TTNtv. During a median follow‐up of 4.4 years, no significant difference in death and heart transplantation between the two groups was observed. However, more HF events occurred in TTNtv carriers than in non‐carriers (*P* = 0.006). Multivariable analyses showed that TTNtv were associated with an increased risk of HF events independent of sex, age, and baseline cardiac function (hazard ratio: 3.25, 95% confidence interval: 1.50‐7.01, *P* = 0.003). Sensitivity analysis excluding non‐A‐band TTNtv yielded similar results, but with less strength.

**Conclusions:**

The presence of TTNtv may be a genetic modifier of LVNC and confer a higher risk of HF events among adult patients. Studies of larger cohorts are needed to confirm our findings.

## 1 INTRODUCTION

Left ventricular non‐compaction cardiomyopathy (LVNC), characterized by excessively prominent trabeculations and deep intratrabecular recesses, is classified as a primary genetic cardiomyopathy by the American Heart Association.^1^, ^2^ The clinical presentation of LVNC is highly heterogeneous, ranging from no obvious symptoms to serious complications including heart failure (HF), thromboembolism, and ventricular arrhythmias.^3^ Therefore, it is crucial to identify patients at high risk for these adverse events and implement appropriate treatment to reduce mortality and morbidity.

Titin, encoded by the *TTN* gene, is a giant filament that spans the hemi‐sarcomere of striated muscle. It plays important roles in sarcomeric integrity, signal transmission, passive stiffness, and contraction regulation.^4^ Although a missense variant of *TTN* has been shown to be associated with a penetrant cardiomyopathy with features of LVNC,^5^ the significance of most missense variants remains unclear.^6^ However, titin‐truncating variants (TTNtv) have been recognized as the most common genetic cause of dilated cardiomyopathy (DCM) and appear to modify the phenotype of hypertrophic cardiomyopathy.^6^, ^7^ Here, we hypothesized that TTNtv might also act as a secondary modifier rather than a primary factor in the context of LVNC. Thus, we conducted this study to investigate the prevalence of TTNtv, and their correlations with clinical manifestations and long‐term prognosis in a Chinese cohort of patients with LVNC.

## 2 METHODS

### 2.1 Study design and subjects

Data were obtained from a cohort of patients with LVNC that has already been described.^8^ LVNC was diagnosed based on echocardiographic or cardiac magnetic resonance findings in accordance with the criteria of Jenni et al or Petersen et al, respectively.^9^, ^10^ Patients were eligible for the study if they had LVNC and were willing to undergo genetic testing and follow‐up.

The study complied with the principles of the Declaration of Helsinki and was approved by the Ethics Committee of Fuwai Hospital. Written informed consent was obtained from all participants.

### 2.2 *TTN* sequencing

Peripheral venous blood samples of the patients were collected for the extraction of genomic DNA. The coding exons and flanking 10‐bp intronic regions of *TTN* as well as 71 other cardiomyopathy‐related genes were comprehensively screened by targeted resequencing. The mean depth of LVNC samples was more than 400× with coverage of more than 99.7%.

Variants were described in accordance with the guidelines for mutation nomenclature of the Human Genome Variation Society (http://www.hgvs.org/). TTNtv were defined as variants that are predicted to yield a truncated form of titin if transcribed and translated, including nonsense, frameshift, and canonical splicing variants. Variants were excluded if their minor allele frequency was ≥0.05% among East Asians in the Genome Aggregation Database.^11^ Sanger sequencing was used to confirm the presence of TTNtv.

### 2.3 Follow‐up and clinical outcomes

Outcome data were obtained through a telephone interview or clinic visit. The last follow‐up was performed in April 2018. The primary endpoint was a composite of all‐cause death and heart transplantation. The secondary endpoint was HF events, a composite of HF‐related death, heart transplantation, and HF hospitalization. HF‐related death was defined as death preceded by symptoms of HF lasting >1 hour. HF hospitalization was defined as a hospital stay of >24 hours with a primary diagnosis of HF during the follow‐up.

### 2.4 Statistical analysis

Categorical variables are presented as frequency and percentage, and continuous variables are expressed as median (interquartile range). Differences in participant characteristics were compared by Pearson's *χ* ^2^ test or Fisher's exact test for categorical variables, and by independent‐sample *t* tests or Mann‐Whitney *U* tests for continuous variables. Survival curves were constructed by the Kaplan‐Meier method and compared by the log‐rank test. Univariable and multivariable Cox proportional hazards regressions were performed to calculate the hazard ratio (HR) and 95% confidence interval (CI), and to evaluate the association between TTNtv and clinical outcomes. Covariates included in the multivariable model were age, sex, and New York Heart Association functional class III/IV at baseline. Differences were considered significant if the two‐sided *P*‐value was <0.05. Sensitivity analyses excluding all non‐A‐band TTNtv were performed to reduce the confounding due to a position related effect of TTNtv. All analyses were performed with SPSS version 22.0 software (IBM Corp., Armonk, New York).

## 3 RESULTS

### 3.1 Study population and genetic findings

A total of 83 adult patients were included in the study, of which 58 (69.9%) were male and the mean age at enrollment was 44 years (Table 1). A total of 13 TTNtv were identified in 13 (15.7%) patients, with 9 variants in the A‐band of titin (Table S1 in Supporting Information). There were no multiple TTNtv carriers. Among the 13 carriers of TTNtv, 2 patients (15.4%) also carried a probably pathogenic variant in other cardiomyopathy‐related genes (Table S2).

**Table 1:** Baseline characteristics of TTNtv carriers and non‐carriers

Characteristics	All patients (n = 83)	TTNtv carriers (n = 13)	Non‐carriers (n = 70)	*P‐*value
Age at enrollment, year	44.0 (34.0‐55.0)	44.0 (29.5‐50.0)	44.5 (34.0–55.0)	0.599
Age of onset, year	40.0 (28.0‐51.0)	36.0 (26.5‐47.5)	41.5 (30.8‐51.3)	0.304
Male, n (%)	58 (69.9)	11 (84.6)	47 (67.1)	0.207
Family history of cardiomyopathy, n (%)	11 (13.3)	1 (7.7)	10 (14.3)	1.000
NYHA class III/IV, n (%)	39 (47.0)	6 (46.2)	33 (47.1)	1.000
LVNC subtypes^a^				0.242
Benign LVNC	17 (20.5)	0 (0.0)	17 (24.3)	
LVNC with arrhythmias	42 (50.6)	8 (61.5)	34 (48.6)	
Dilated LVNC	16 (19.3)	5 (38.5)	11 (15.7)	
Hypertrophic LVNC	4 (4.8)	0 (0.0)	4 (5.7)	
Hypertrophic dilated LVNC	0 (0.0)	0 (0.0)	0 (0.0)	
Restrictive LVNC	1 (1.2)	0 (0.0)	1 (1.4)	
Right ventricular or biventricular LVNC	1 (1.2)	0 (0.0)	1 (1.4)	
LVNC with congenital heart disease	2 (2.4)	0 (0.0)	2 (2.9)	
Comorbidities				
Coronary artery disease, n (%)	9 (10.8)	0 (0.0)	9 (12.9)	0.342
Hypertension, n (%)	13 (15.7)	0 (0.0)	13 (18.6)	0.205
Diabetes, n (%)	7 (8.4)	1 (7.7)	6 (8.6)	1.000
Hyperlipidemia, n (%)	14 (16.9)	4 (30.8)	10 (14.3)	0.219
Atrial fibrillation, n (%)	15 (18.1)	4 (30.8)	11 (15.7)	0.239
Echocardiography				
LVEDD, mm	62.0 (54.8‐70.0)	67.0 (62.5‐72.0)	61.0 (54.0‐70.0)	0.115
LAD, mm	41.5 (35.0‐48.0)	42.0 (34.5‐51.0)	41.0 (35.0–48.0)	0.598
LVEF, %	38.5 (30.8‐52.3)	32.2 (24.5‐42.5)	40.0 (31.0‐54.0)	0.137

Abbreviations: LAD, left atrial diameter; LVEDD, left ventricular end‐diastolic dimension; LVEF, left ventricular ejection fraction; LVNC, left ventricular non‐compaction cardiomyopathy; NYHA, New York Heart Association; TTNtv, titin‐truncating variants.

a Classified according to the review by Towbin et al.^1^

### 3.2 Genotype‐phenotype correlation at baseline

There were no significant differences between TTNtv carriers and non‐carriers in terms of demographic data, LVNC subtypes, comorbidities, cardiac function, or echocardiographic findings (Table 1).

### 3.3 Genotype‐phenotype correlation for outcomes

**Figure 1:**
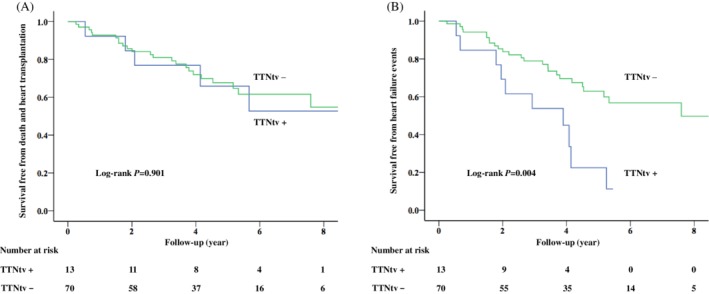
Survival curves of the primary endpoint (A) and the secondary endpoint (B). TTNtv, titin‐truncating variant

During a median follow‐up of 4.4 (2.8‐6.2) years, 28 (33.7%) patients reached the primary endpoints and 35 (42.2%) experienced HF events (Table 2). There were significantly more HF events and HF hospitalizations in TTNtv carriers than in non‐carriers (*P* = 0.006 and 0.003, respectively). No significant differences in other endpoints were observed between TTNtv carriers and non‐carriers. Univariable and multivariable analyses found that the New York Heart Association functional class III/IV at baseline was a strong predictor of the primary and secondary endpoints (Table 3). In addition, the presence of TTNtv was significantly associated with an increased risk of HF events independently of sex, age, and baseline cardiac function (HR: 3.25, 95% CI: 1.50‐7.01, *P* = 0.003). Survival curves of the primary and secondary endpoints are shown in Figure 1.

**Table 2:** Incidence of primary and secondary endpoints in TTNtv carriers and non‐carriers

	All patients (n = 83)	TTNtv carriers (n = 13)	Non‐carriers (n = 70)	*P‐*value
Primary endpoint				
Death and heart transplantation, n (%)	28 (33.7)	5 (38.5)	23 (32.9)	0.754
All‐cause death, n (%)	24 (28.9)	3 (23.1)	21 (30.0)	0.748
Heart transplantation, n (%)	4 (4.8)	2 (15.4)	2 (2.9)	0.114
Secondary endpoint				
HF events, n (%)	35 (42.2)	10 (76.9)	25 (35.7)	0.006
HF‐related death, n (%)	19 (67.8)	3 (23.1)	16 (22.9)	1.000
HF hospitalization, n (%)	31 (37.3)	10 (76.9)	21 (30.0)	0.003

Abbreviations: HF, heart failure; TTNtv, titin‐truncating variants.

**Table 3:** Univariable and multivariable analysis of predictors for primary and secondary endpoints

	Univariable			Multivariable*		
	HR	95% CI	*P‐*value	HR	95% CI	*P‐*value
Death and heart transplantation						
TTNtv	1.06	0.40‐2.80	0.901	–	–	–
Male sex	1.09	0.48‐2.47	0.841	–	–	–
Age	1.00	0.99‐1.03	0.461	–	–	–
NYHA class III/IV	2.93	1.29‐6.68	0.010	2.94	1.26‐6.86	0.013
Heart failure events						
TTNtv	2.88	1.37‐6.07	0.005	3.25	1.50–7.01	0.003
Male sex	1.17	0.56‐2.45	0.669	–	–	–
Age	1.01	0.99‐1.03	0.385	–	–	–
NYHA class III/IV	3.58	1.71‐7.47	0.001	3.89	1.83‐8.28	<0.001

Abbreviations: CI, confidence interval; HR, hazard ratio; NYHA, New York Heart Association; TTNtv, titin‐truncating variants.

* Items with *P* < 0.05 in univariable analysis was then included in the calculation of multivariable HR and 95% CI.

### 3.4 Sensitivity analysis

A‐band TTNtv are suggested to have higher penetrance than variants of other regions in the context of DCM.^12^ Therefore, sensitivity analyses were performed after the exclusion of non‐A‐band TTNtv. A total of nine A‐band TTNtv were detected in nine patients, who had larger left ventricular end‐diastolic dimension and lower left ventricular ejection fraction (Tables S1 and S3). During the follow‐up, significantly more HF events and HF hospitalizations occurred in A‐band TTNtv carriers than in non‐carriers (Table S4). After adjustment, a marginal association was found between TTNtv and HF events (HR: 2.35, 95% CI: 0.99‐5.58, *P* = 0.052, Table S5). Notably, five of the nine patients with A‐band TTNtv had an additional diagnosis of DCM (dilated LVNC, Table S2).

## 4 DISCUSSION

In a cohort of 83 adult patients with LVNC, 13 TTNtv were identified in 13 participants, including 9 TTNtv in the A‐band region of titin. No significant phenotypic differences were found between TTNtv carriers and non‐carriers at baseline. During the follow‐up, the presence of TTNtv was associated with an increased risk of an HF event after adjustment for sex, age, and baseline cardiac function. Sensitivity analysis excluding non‐A‐band TTNtv yielded similar results, but with less significance.

As the most prevalent genetic cause of DCM, TTNtv account for 20% to 25% of familial cases.^13^ Interestingly, it has been reported that TTNtv are also detected in about 10% to 15% of patients with LVNC,^14^, ^15^ although no causal association between TTNtv and LVNC has been identified. Consistent with these studies, 15% of the patients in our study were found to carry TTNtv. The reason why TTNtv are enriched in LVNC patients compared with the general population (~1%) has yet to be clarified.^12^ One possible explanation is the existence of a syndrome with overlap of LVNC and DCM, as shown by our study where nearly 40% of TTNtv carriers were diagnosed with both LVNC and DCM.

It has been proposed that there is a correlation between TTNtv and phenotype in the context of LVNC. In a cohort study by Sedaghat‐Hamedani et al,^14^ patients with LVNC and TTNtv showed unfavorable outcomes including arrhythmias and heart transplantation. However, no definitive conclusions could be made on this issue due to the small number of participants. In another larger cohort study of LVNC patients by van Waning et al.,^15^ TTNtv were found to confer a higher risk of left ventricular systolic dysfunction, which is a precursor or feature of HF. Similarly, in our study, TTNtv were associated with an increased risk of HF events, which provided additional evidence for the genotype‐phenotype correlation, and for the hypothesis that TTNtv could modify the clinical outcomes of LVNC.

TTNtv may contribute to a substantially increased risk of HF events through several possible mechanisms. First, TTNtv can cause decreased and disorganized sarcomeres in zebrafish heart, human cardiomyocytes, and cardiac microtissues.^16^, ^17^, ^18^ Such defects have been proven to diminish contractile performance in rodents and humans,^12^, ^19^ which can eventually result in a higher risk of HF events. Second, common metabolic and signaling perturbations have been seen in rodent and human hearts with TTNtv, including a shift towards glycolytic metabolism and pronounced cardiac alterations in mitochondrial function,^12^, ^20^ which can be maladaptive responses to stress and lead to susceptibility to HF. Third, TTNtv have been associated with ventricular and atrial arrhythmias,^20^, ^21^ which can also contribute to an increased risk of HF events.^22^ These adverse effects can be even more prominent in the context of LVNC, in which there was originally supposed to be a higher risk of HF.

A meta‐analysis showed that A‐band TTNtv had larger odds ratios than other TTNtv, suggesting position‐dependent effects on the penetrance of TTNtv in DCM.^12^ In our study, the association between TTNtv and HF events remained irrespective of whether non‐A‐band TTNtv were included. Patients with A‐band TTNtv had worse cardiac function than non‐carriers, but such differences were not observed with the addition of non‐A‐band TTNtv. Therefore, we can speculate that both A‐band and non‐A‐band TTNtv can modify the phenotype of LVNC, with stronger effects by the former.

It has been recognized that nearly 40% of adult patients with LVNC could experience HF events.^23^ Consistent with this, 42% of the patients in our study suffered HF events during follow‐up, underlining the high risk of HF‐related adverse outcomes associated with this disease. However, risk stratification has been challenging in these patients due to a lack of specific prognosticators. In this regard, our findings that TTNtv confer an increased risk of HF events are important because TTNtv can be informative in risk assessment. For example, patients with TTNtv need close follow‐up and may benefit from the early initiation of anti‐HF therapy.

Some limitations to our study should be noted. First, it was an observational study, which could suffer from residual confounding. Second, all participants were of Chinese Han ancestry from a single center, which might limit the generalizability of our findings. Third, the sample size was relatively small and insufficient to draw definite conclusions. Further study with a larger sample size is needed to confirm our findings. Fourth, functional models to corroborate our findings are still lacking.

## 5 CONCLUSIONS

In a Chinese cohort of patients with LVNC, the presence of TTNtv was found to be associated with an increased risk of HF events independent of sex, age, and baseline cardiac function. The identification of TTNtv may contribute to overall risk assessment in LVNC.

## CONFLICT OF INTEREST

The authors declare that they have no conflicts of interest.

## Supporting information


**TABLE S1** Titin‐truncating variants detected in patients with left ventricular non‐compaction cardiomyopathy
**TABLE S2.** Genotypic background and clinical characteristics of patients with TTNtv
**TABLE S3.** Baseline characteristics of patients with and without A‐band TTNtv
**TABLE S4.** Incidence of primary and secondary endpoints in patients with and without A‐band TTNtv
**TABLE S5.** Univariable and multivariable analysis for outcomes in patients with and without A‐band TTNtv
