# Supplementary material for: Titin‐truncating variants are associated with heart failure events in patients with left ventricular non‐compaction cardiomyopathy
Source: Clin Cardiol. 2019 Apr 16;42(5):530–5. doi: 10.1002/clc.23172 (PMC6523007; doi:10.1002/clc.23172)
Supplement: Supplementary file 1 — TABLE S1 Titin‐truncating variants detected in patients with left ventricular non‐compaction cardiomyopathy TABLE S2. Genotypic background and clinical characteristics of patients with TTNtv TABLE S3. Baseline characteristics of patients with and without A‐band TTNtv TABLE S4. Incidence of primary and secondary endpoints in patients with and without A‐band TTNtv TABLE S5. Univariable and multivariable analysis for outcomes in patients with and without A‐band TTNtv [file CLC-42-530-s001.docx]

**Supplemental material**

**Table S1.** Titin-truncating variants detected in patients with left ventricular noncompaction cardiomyopathy

| Genomic start position_hg19 | Protein region | Variant type | Transcript | Transcript effect | Protein effect | GnomAD_ALL_AF^*^ | GnomAD_EAS_AF^†^ | dbSNP | Patient ID |
| --- | --- | --- | --- | --- | --- | --- | --- | --- | --- |
| 179635290 | I-Band | Frameshift | NM_001267550 | c.8228delA | p.N2743fs | NA | NA | . | 86 |
| 179603867 | I-Band | Splicing | NM_001267550 | c.14092+1G>A |  | NA | NA | . | 92 |
| 179511789 | I-Band | Splicing | NM_001267550 | c.40222+2->AATA |  | NA | NA | . | 104 |
| 179478480 | A-Band | Nonsense | NM_001267550 | c.C49530A | p.Y16510X | NA | NA | . | 120 |
| 179471932 | A-Band | Frameshift | NM_001267550 | c.53396_53397insGCTTT | p.F17799fs | NA | NA | . | 34 |
| 179465800 | A-Band | Frameshift | NM_001267550 | c.55829_55830del | p.P18610fs | NA | NA | . | 81 |
| 179456446 | A-Band | Frameshift | NM_001267550 | c.60099dupA | p.E20034fs | NA | NA | . | 79 |
| 179454910 | A-Band | Frameshift | NM_001267550 | c.61538_61541del | p.E20513fs | NA | NA | . | 73 |
| 179454590 | A-Band | Nonsense | NM_001267550 | c.C61862A | p.S20621X | NA | NA | . | 100 |
| 179442627 | A-Band | Splicing | NM_001267550 | c.68528-2A>T | . | NA | NA | rs779485172 | 29 |
| 179433843 | A-Band | Nonsense | NM_001267550 | c.C77016G | p.Y25672X | NA | NA | . | 93 |
| 179412788 | A-Band | Nonsense | NM_001267550 | c.A93565T | p.K31189X | NA | NA | . | 45 |
| 179398283 | M-Band | Frameshift | NM_001267550 | c.103058delA | p.H34353fs | NA | NA | . | 102 |

^*^ Minor allele frequencies of variants among total population in the Genome Aggregation Database (*Nature*. 2016;536: 285-91)

**^†^** Minor allele frequencies of variants among East Asians in the Genome Aggregation Database (*Nature*. 2016;536: 285-91)

**Table S2.** Genotypic background and clinical characteristics of patients with TTNtv

| Patient ID | Sex | Age at enrollment | Variant | A-band TTNtv | LVNC subtype | Outcome |
| --- | --- | --- | --- | --- | --- | --- |
| 29 | Male | 51 | *TTN* c.68528-2A>T | Yes | LVNC with arrhythmias | HF hospitalization |
| 34 | Male | 18 | *TTN* c.53396_53397insGCTTT | Yes | Dilated LVNC | HF hospitalization, HF-related death |
| 45 | Male | 39 | *TTN* c.A93565T | Yes | LVNC with arrhythmias | HF hospitalization, HF-related death |
| 73 | Male | 78 | *TTN* c.61538_61541del | Yes | LVNC with arrhythmias | HF hospitalization, HF-related death |
| 79 | Female | 36 | *TTN* c.60099dupA | Yes | Dilated LVNC | HF hospitalization |
| 81 | Male | 30 | *TTN* c.55829_55830del | Yes | Dilated LVNC |  |
| 86 | Male | 65 | *TTN* c.8228delA;  *MYBPC3* c.1377delC | No | LVNC with arrhythmias | HF hospitalization |
| 92 | Female | 36 | *TTN* c.14092+1G>A;  *MYBPC3* c.1352_1379del | No | LVNC with arrhythmias | HF hospitalization |
| 93 | Male | 28 | *TTN* c.C77016G | Yes | LVNC with arrhythmias | HF hospitalization, HT |
| 100 | Male | 27 | *TTN* c.C61862A | Yes | Dilated LVNC | HF hospitalization |
| 102 | Male | 23 | *TTN* c.103058delA | No | LVNC with arrhythmias | HF hospitalization, HT |
| 104 | Male | 26 | *TTN* c.40222+2->AATA | No | LVNC with arrhythmias |  |
| 120 | Male | 44 | *TTN* c.C49530A | Yes | Dilated LVNC |  |

HF, heart failure; HT, heart transplantation; LVNC, left ventricular noncompaction cardiomyopathy; TTNtv, titin-truncating variants.

**Table S3.** Baseline characteristics of patients with and without A-band TTNtv

| Characteristics | All patients (n=83) | A-band TTNtv carriers (n=9) | Non-carriers (n=74) | *P‒*value |
| --- | --- | --- | --- | --- |
| Age at enrollment, year | 44.0 (34.0‒55.0) | 44.0 (36.0‒50.0) | 44.0 (33.8‒55.0) | 0.855 |
| Age of onset, year | 40.0 (28.0‒51.0) | 36.0 (27.5‒47.5) | 40.5 (29.5‒51.3) | 0.517 |
| Male, n (%) | 58 (69.9) | 8 (88.9) | 50 (67.6) | 0.188 |
| Family history of cardiomyopathy, n (%) | 11 (13.3) | 1 (11.1) | 10 (13.5) | 1.000 |
| NYHA class III/IV, n (%) | 39 (47.0) | 5 (55.6) | 34 (45.9) | 0.728 |
| Comorbidities |  |  |  |  |
| Coronary artery disease, n (%) | 9 (10.8) | 0 (0.0) | 9 (12.2) | 0.587 |
| Hypertension, n (%) | 13 (15.7) | 0 (0.0) | 13 (17.6) | 0.342 |
| Diabetes, n (%) | 7 (8.4) | 1 (11.1) | 6 (8.1) | 0.567 |
| Hyperlipidemia, n (%) | 14 (16.9) | 4 (44.4) | 10 (13.5) | 0.040 |
| Atrial fibrillation, n (%) | 15 (18.1) | 2 (22.2) | 13 (17.6) | 0.663 |
| Echocardiography |  |  |  |  |
| LVEDD, mm | 62.0 (54.8‒70.0) | 68.0 (65.5‒72.0) | 61.0 (52.5‒70.0) | 0.020 |
| LAD, mm | 41.5 (35.0‒48.0) | 42.0 (36.0‒51.0) | 41.0 (35.0‒48.0) | 0.533 |
| LVEF, % | 38.5 (30.8‒52.3) | 28.0 (21.0‒36.6) | 40.0 (31.5‒55.5) | 0.006 |

LAD indicates left atrial diameter; LVEDD, left ventricular end-diastolic dimension; LVEF, left ventricular ejection fraction; NYHA, New York Heart Association; TTNtv, titin-truncating variants.

**Table S4.** Incidence of primary and secondary endpoints in patients with and without A-band TTNtv

|  | All patients (n=83) | A-band TTNtv carriers  (n=9) | Non-carriers  (n=74) | *P-*value |
| --- | --- | --- | --- | --- |
| Primary endpoint |  |  |  |  |
| Death and heart transplantation, n (%) | 28 (33.7) | 4 (44.4) | 24 (32.4) | 0.478 |
| All-cause death, n (%) | 24 (28.9) | 3 (33.3) | 21 (28.4) | 0.714 |
| Heart transplantation, n (%) | 4 (4.8) | 1 (11.1) | 3 (4.1) | 0.374 |
| Secondary endpoint |  |  |  |  |
| Heart failure event, n (%) | 35 (42.2) | 7 (77.8) | 28 (37.8) | 0.032 |
| Heart failure-related death, n (%) | 19 (22.9) | 3 (33.3) | 16 (21.6) | 0.421 |
| Heart failure hospitalization, n (%) | 31 (37.3) | 7 (77.8) | 24 (32.4) | 0.012 |

TTNtv indicate titin-truncating variants.

**Table S5.** Univariable and multivariable analysis for outcomes in patients with and without A-band TTNtv

|  | Univariable | | | Multivariable* | | |
| --- | --- | --- | --- | --- | --- | --- |
|  | HR | 95% CI | *P-*value | HR | 95% CI | *P-*value |
| Death and heart transplantation |  |  |  |  |  |  |
| A-band TTNtv | 1.13 | 0.39‒3.25 | 0.828 | ‒ | ‒ | ‒ |
| Male sex | 1.09 | 0.48‒2.47 | 0.841 | ‒ | ‒ | ‒ |
| Age | 1.01 | 0.99‒1.03 | 0.461 | ‒ | ‒ | ‒ |
| NYHA class III/IV | 2.93 | 1.29‒6.68 | 0.010 | 2.97 | 1.26‒6.98 | 0.013 |
| Heart failure event |  | | |  | | |
| A-band TTNtv | 2.54 | 1.10‒5.84 | 0.029 | 2.35 | 0.99‒5.58 | 0.052 |
| Male sex | 1.17 | 0.56‒2.45 | 0.669 | ‒ | ‒ | ‒ |
| Age | 1.01 | 0.99‒1.03 | 0.385 | ‒ | ‒ | ‒ |
| NYHA class III/IV | 3.58 | 1.71‒7.47 | 0.001 | 3.57 | 1.67‒7.64 | 0.001 |

CI indicates confidence interval; HR, hazard ratio; NYHA, New York Heart Association; TTNtv, titin-truncating variants.

*Items with *P*<0.05 in univariable analysis was then included in the calculation of multivariable HR and 95% CI.
